# The effect of P2Y12 receptor inhibition in chronic inflammatory pain

**DOI:** 10.1186/2193-1801-4-S1-P2

**Published:** 2015-06-12

**Authors:** Katinka Bekő, Ádám Dénes, Gergely Horváth, Beáta Sperlágh

**Affiliations:** Laboratory of Molecular Pharmacology, Institute of Experimental Medicine, Hungarian Academy of Sciences, Hungary; János Szentágothai School of Neurosciences, Semmelweis University School of Ph.D Studies, Hungary; Laboratory of Molecular Neuroendocrinology, Institute of Experimental Medicine, Hungarian Academy of Sciences, Budapest, Hungary

**Keywords:** inflammation, pain, purin

Previous studies of our group indicate that genetic deletion and pharmacological antagonism of P2Y12 receptors alleviate mechanical allodynia in acute inflammatory pain. In this project we investigated the role of P2Y12 receptors in chronic inflammation. For this study we have introduced a CFA-model in wild type and P2Y12R gene deficient (P2ry12-/-) mice. By the injection of Complete Freund’s Adjuvant (CFA) in the plantar surface of the right hind paw, we could induce local inflammation persisting at least 14 days. During this 2 week period, mechanical sensitivity was evaluated at several time points using dynamic plantar von Frey aesthesiometer. Mechanical allodynia could be observed 3 days after CFA injection. In P2Y12 receptor gene deficient mice the decline in the paw withdrawal threshold (PWT) was significantly lower than in wild type mice. Both intrathecal and intraplantar administration of PSB-0739, a selective and potent P2Y12 receptor antagonist had robust antihyperalgesic effect in wild type mice, whereas treatment did not affect the PWT in the P2ry12-/- group. Intraperitoneal injection of A-803467, a potent and selective NaV1.8 sodium channel blocker evoked antihyperalgesic effect similar to the PSB-0739. When these two compounds added together, no additive effect was observed, i.e. A-803467 occluded the effect of PSB-0739. To investigate whether P2Y12 receptors on thrombocytes contribute to this effect, we used anti-mouse CD41 antibodies to induce depletion of platelets. Mice were submitted to intraperitoneal injection of this antibody at the end of the first week and measurement of the mechanical allodynia was performed on the second week. Our findings indicate that P2Y12 receptor inhibition is a potential therapeutic approach in chronic inflammatory pain, although its exact mechanism of action needs further investigation.

